# SeedStor: A Germplasm Information Management System and Public Database

**DOI:** 10.1093/pcp/pcx195

**Published:** 2017-12-08

**Authors:** RSP Horler, AS Turner, P Fretter, M Ambrose

**Affiliations:** 1 Germplasm Resources Unit, John Innes Centre, Colney Lane, Norwich NR4 7UH, UK; 2 Computing Infrastructure for Science, Norwich Bioscience Institutes Partnership, Colney Lane, Norwich NR4 7UH, UK; 3 Norwich Bioscience Institutes Partnership, Colney Lane, Norwich NR4 7UH, UK

**Keywords:** Database, Germplasm, Information management, Pea, Seed Bank, Small grain cereals

## Abstract

SeedStor (https://www.seedstor.ac.uk) acts as the publicly available database for the seed collections held by the Germplasm Resources Unit (GRU) based at the John Innes Centre, Norwich, UK. The GRU is a national capability supported by the Biotechnology and Biological Sciences Research Council (BBSRC). The GRU curates germplasm collections of a range of temperate cereal, legume and Brassica crops and their associated wild relatives, as well as precise genetic stocks, near-isogenic lines and mapping populations. With >35,000 accessions, the GRU forms part of the UK’s plant conservation contribution to the Multilateral System (MLS) of the International Treaty for Plant Genetic Resources for Food and Agriculture (ITPGRFA) for wheat, barley, oat and pea. SeedStor is a fully searchable system that allows our various collections to be browsed species by species through to complicated multipart phenotype criteria-driven queries. The results from these searches can be downloaded for later analysis or used to order germplasm via our shopping cart. The user community for SeedStor is the plant science research community, plant breeders, specialist growers, hobby farmers and amateur gardeners, and educationalists. Furthermore, SeedStor is much more than a database; it has been developed to act internally as a Germplasm Information Management System that allows team members to track and process germplasm requests, determine regeneration priorities, handle cost recovery and Material Transfer Agreement paperwork, manage the Seed Store holdings and easily report on a wide range of the aforementioned tasks.

## Introduction

Ready and reliable access to well-curated diverse germplasm resources is essential to underpinning advances in understanding plant sciences and providing the source of novel and adaptive traits for crop improvement. The Germplasm Resources Unit (GRU) is focused on the curation and provision of plant germplasm specializing in small grain cereals and grain legumes, and is now starting to accept resources in oilseed rape. As with all gene banks, the GRU is rich in data resources associated with the collections.

The Seed Store acts as the UK repository for wheat, barley, oats, peas and broad beans under the UK contribution to the International Treaty for Plant Genetic Resources for Food and Agriculture (ITPGRFA). We also hold and distribute seeds from a range of precise genetic stocks and mapping populations as well as the original population samples from the Waktins Collection and single seed descent (SSD) materials derived from this collection (Miller 2001, Wingen et al. 2014). The service we offer enables researchers to access a wide range of cultivars for research use and has been acknowledged in multiple publications (Jing et al. 2010, Casebow et al. 2016, Prins et al. 2016, Orsenigo et al. 2017).

In addition to collections we hold that have been collected from geographically diverse locations over many years, we also hold stocks of seeds that have been created as output from various research projects. This forms part of the data and materials sharing component of many research grants, and thus submission to a recognized gene bank such as the GRU is a simple method to achieve this. A recent addition of this type to the GRU is the wheat TILLING population created by ethyl methanesulfonate (EMS)-mutagenized SSD lines of the Cadenza and Kronos cultivars (Uauy et al. 2009, Krasileva et al. 2017).

The GRU engages with a wide range of stakeholder groups. By far the largest is the research community, which accounts for 65% of all enquiries. Commercial and state breeding requests account for 10% and the remaining 25% include educational establishments (primary, secondary and tertiary levels), commercial food companies through to hobby growers.

In 2012, the GRU became a national capability supported by the Biotechnology and Biological Sciences Research Council (BBSRC) and received additional fixed term staff resources to develop an integrated information management system that would service the operational needs of the GRU and facilitate access to information and services to our customer base. It was to fulfill these needs that SeedStor was developed. The SeedStor project was jointly managed and developed by the GRU and Computing infrastructure for Science (CiS) group within the John Innes Centre (JIC) and Norwich Biosciences Institute Partnership (NBIP), respectively. The developmental project officer was officially managed by a member of the GRU but embedded within CiS. This helped ensure that the software development was professionally monitored and harmonized to the correct operational practises of CiS and that corporate knowledge of the system was present within CiS to continue to support the software and its future development beyond the life span of the funding period.

## Description of SeedStor

The current release of SeedStor is version 1.06 and includes 11 of our collections or about 35,000 different accessions. For each accession, we hold a definitive record of passport data together with records for one or more additional datasets including phenotype, taxonomy, pedigree and expedition data. Wherever possible, this has been achieved with reference to the accepted data format (FAO/IPGRI 2001). The most critical aspect of our records is the reliable stock data of that accession of which there might be multiple records from different sources from medium scale field regeneration through to glasshouse regeneration of a few plants at a time under controlled conditions.

## Search Interface

The main functionality of SeedStor from the external user perspective is provided by the ability to search across our publicly available collections for materials that may be of interest and place an order. The interface has been designed and revised with input from targeted individuals from across our stakeholder groups of which the research and plant breeding are the largest sectors through to hobbyists interested in growing material for thatching, brewing, baking, education or simply for historic accuracy in various heritage farms.

**Fig. 1:**
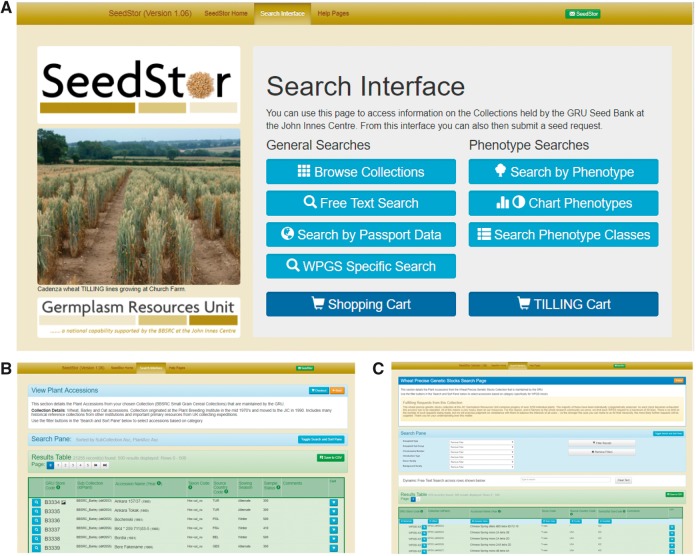
SeedStor public database. (A) The primary search page which provides access to the different routes to query the SeedStor Database. (B) Search results page from the Browse Collections query method. The results can be further filtered, sorted and exported using functions provided on the page. (C) The WPGS (wheat precise genetic stocks) have specific characteristics that are integral to this collection, and this search method allows users to query the database for these traits.

There are many different methods to interrogate SeedStor to take advantage of the wide range of data held on our material. These are accessed via our Search Interface (Fig. 1A) as are the shopping carts for checking out an order once completed.

### Browse collections

As our material is held within specific collections, the easiest method to peruse through is by looking at the stocks on a collection basis using the ‘Browse Collection’ search. This search also allows further filtering of collections based on taxonomy, sowing season and country of origin as well as to sort the resulting accessions by the same criteria (Fig. 1B). Icons appear adjacent to the primary identifier (GRU Store Code) to indicate when an accession has photo and expedition data. Full details for a specific accession can be viewed by clicking on the left-hand Magnifying Glass button or the line added to the shopping cart by selecting the button on the right-hand side of each entry. It is also possible to download the resulting Accessions List to a CSV file for future perusal.

### Free text search

For those who are looking for a specific accession, there is a simple free text search on the home page but within the search interface there is a more powerful ‘Free Text Search’ that allows text-based searches against specified fields from Passport, Taxonomy, Phenotype and Collection information using standard, fuzzy and wildcard searches with any or all terms.

### Search by passport data

This functionality enables users to search across the different collections for specific taxa, sowing season or country of origin. The search page is designed to handle simple single criteria searches and more complex multifaceted search criteria from within the standard Passport data held on each accession.

### WPGS search

While most of the collections we hold are based on expeditions that have collected accessions in the wild and commerce, we also hold several collections that have been developed in the lab and as such require specific regeneration methods to ensure the consistency of the accessions. One such example of this is the Wheat Precise Genetic Stocks Collection (WPGSC); these specialist lines developed in the 1980s and still in high demand today require much more care in their propagation due to the unstable nature of their chromosomal constitution. The ‘WPGS Specific Search’ functions (Fig. 1C) allow users to select based on criteria specific to this collection including aneuploid type, chromosome number carrying the translocation/deletion/addition as well as background and donor variety.

## Search Interface: Phenotypes

SeedStor offers three different mechanisms to query the phenotype data held on the accessions. The first is a comprehensive search that allows users to combine different phenotypic traits to identify accessions that meet all, or some, of their criteria requirements. The second is a graphical representation to show the distribution of a trait across all accessions held. The final method is one that allows users to query phenotypes within a specific taxonomy or phenotype class, e.g. flower-specific traits or disease traits.

### Search by phenotype

**Fig. 2:**
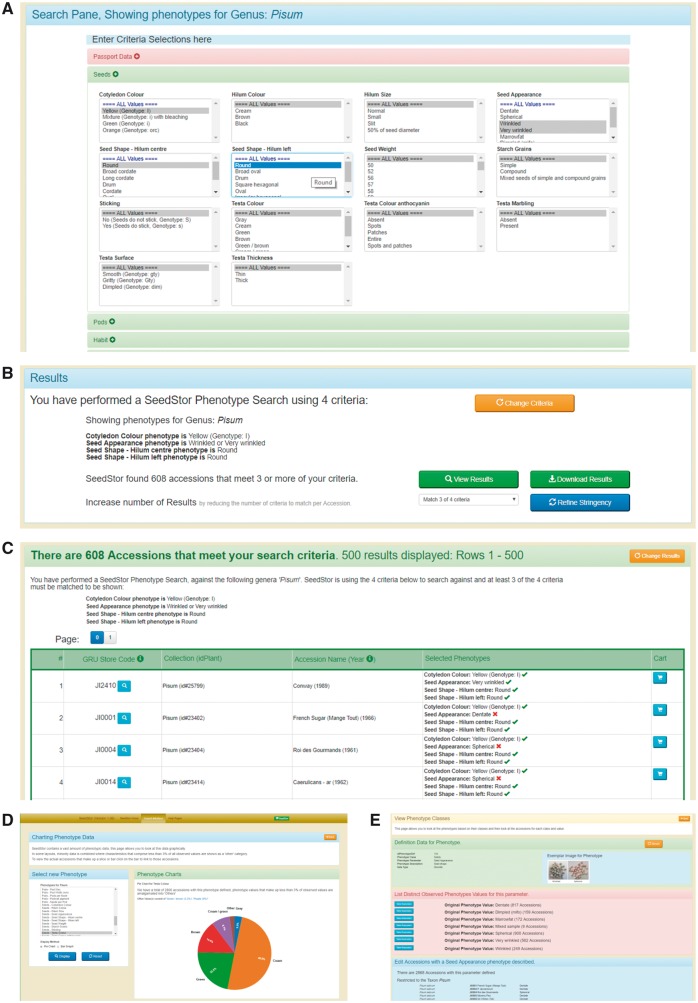
Phenotype searches available via SeedStor. (A) The ‘Search by Phenotypes’ function allows users to select different phenotype parameters and observed traits either as sole criteria or as a multipart query. (B) The results then can be viewed on screen or exported as a flat CSV file for later analysis, and in addition the stringency of the search can be adapted to find sufficient material to investigate. (C) When the results are viewed via SeedStor, the search criteria are listed for each accession together with the observed character. (D) A more visual method of investigating specific phenotypes is via the charting tool, which allows users to view the distribution of characters for a specific trait and then, by clicking on the pie slice or bar, view the accessions that match. (E) The final method for looking at phenotype information is to view this as SeedStor records it together with all matching lines, any general information on the trait and any exemplar images for the trait.

After selecting the taxon of interest (as phenotypes are specific to each taxon within SeedStor) it is then possible to select multiple traits and observed values from the presented options (Fig. 2A). Within this, it is also possible to include three passport fields that may be of interest within these phenotype queries namely: Sowing Season, Biological Sample Status and Country of Origin. To aid in selection, phenotypes are grouped into Phenotype Classes and each class is expanded when selected to show the phenotype traits, and thus users can then select those observed traits as required. Traits can be either discrete, for example flower color or seed shape, or continuous, for example plant height or pod length.

Once the different criteria have been selected for each trait of interest, the query can then be triggered via the search button and the results generated. The returned page will present the search criteria, the number of results found (a single accession in the example given) and the level of stringency. The user can then choose to access those results as a downloadable CSV file or onscreen via SeedStor itself (Fig. 2B). The user can also decide to lower the stringency in the hope of identifying more matches to the criteria specified, i.e. accept any accession that meets three of the above four criteria selected; under these rules, 608 accessions are found for our example.

If SeedStor is used to View Results, then the final screen lists those accessions that meet the criteria at the specified stringency level as well as restating the selected criteria (Fig. 2C). Each accession also clearly shows the phenotype(s) observed and whether this is a match or not to the criteria, and can be directly added to the user’s shopping cart or further information on the accession provided by the normal link.

### Chart phenotype

Again, after selecting the taxon of interest, a phenotype can be selected and the associated data plotted as either a bar graph or a pie chart (Fig. 2D). These display the distribution of different characters in an easily visualized form, and the actual accessions can then be listed by clicking on the relevant chart segment.

### Search phenotype classes

Search Phenotype Classes is a tool that takes advantage of the grouping of phenotypes into taxa and classes used by SeedStor to view characteristics. This output includes both total numbers for each observed trait and a list of matching accessions (Fig. 2E). Where we have exemplar images that help define the various phenotype classes, these are shown together with a full descriptor of the phenotype parameter.

## Shopping Cart

**Fig. 3:**
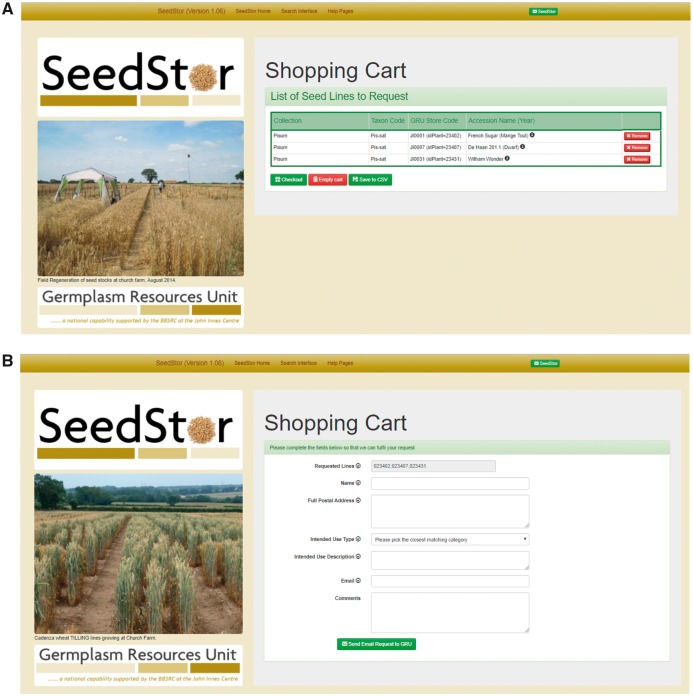
The shopping cart provides the main function of the website, the ability to request materials from the GRU. (A) The first stage is to verify that the lines within the shopping cart are as requested and then move onto checking out and (B) completing the information required so that the GRU can send material to the end user.

The key purpose of the SeedStor database is to provide easy public access to requesting seed stocks for use. All the search functions include the ability to add the desired lines to the shopping cart (Fig. 3A) and, once selection is complete, it is possible to check out the order. The shopping cart will list all the requested lines and then, when the user is ready, they can proceed to a form that will request their details and E-mail the order through to GRU staff (Fig. 3B). This form is specific to the details of the particular collections from which the shopping cart lines have been drawn so the user form asks for delivery information and, when needed, will detail any cost recovery or specific Material Transfer Agreement (MTA) requirements.

## Management functions

For use within the GRU, SeedStor functions as an essential management system and handles the diverse range of requests that come in for both information and seed samples. It also manages all the other operational processes managed by the GRU in maintaining our collections from seed regeneration through to ensuring we are legally compliant with the obligations under the various international treaties and legislation.

**Fig. 4:**
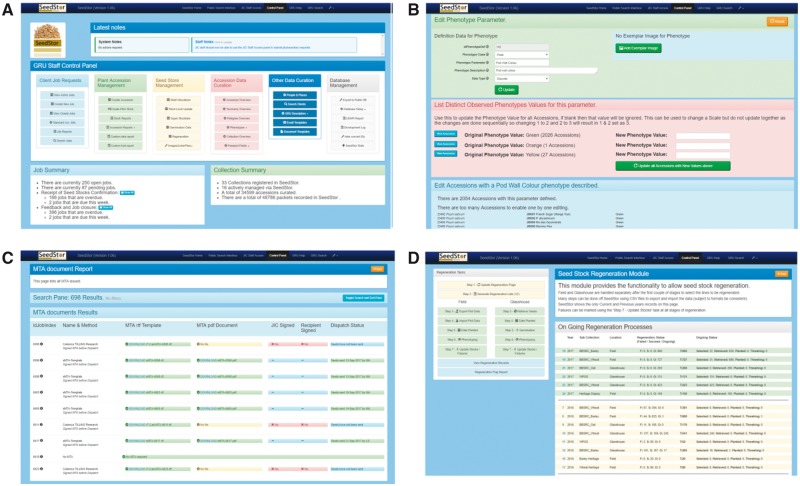
The Seed Store management functions within SeedStor form the major component of the system. It is used via a restricted access Control Panel (A) which also shows the current status overview of job requests and collections. Through the control panel, GRU staff can access functionality for curating new accessions, for example adding and editing phenotypes (B), managing the official documentation needed for the distribution of seed stocks (C) and managing the yearly regeneration of material (D).

The management functions are all accessible via a specific control panel that is restricted to authorized GRU personnel only (Fig. 4A). The security behind this is based on the site-wide LDAP authentication followed by a white list of known users with access controlled by SeedStor itself. The Control panel is split into different functions; each of these acts as a fully configurable module that allows us to control all aspects of the SeedStor database. In this section, we have selected three specific examples of the configuration of SeedStor and how it provides the GRU with a powerful tool in carrying out its function: (i) curation; (ii) MTA templates; and (iii) regeneration.

*Curation.* An important function of the GRU is to ensure the curated information we hold for all our accessions are current, reliable and as expansive as we can achieve. This is made difficult by the vast range of collections that we hold and curate from a wide range of different species, and thus these different collections require different information to be recorded. We solve this by maintaining a core Passport dataset for each accession and then optional aspects on where the material was originally sourced (Expedition data); taxa information is held for all accessions, although some hybrid lines make this additionally complex. The final and most complicated aspect is recording phenotype data; the solution we use is to store these in a database-friendly format using a three-key system of Class, Parameter and Value for storing phenotype data. This allows us to represent vastly different phenotypic characteristics from the wide range of material we hold while using a defined structure to allow the database to be so effectively queried. It also allows us to make wholesale changes when it is necessary to redefine a parameter following changes in the accepted nomenclature (Fig. 4B).

*MTA templates*. All material sent out is released under an MTA that is specific to that collection. We have a responsibility to ensure these are properly documented and auditable, and SeedStor handles this through the job request process and associated templates for each MTA. (The job request process is started when a material request is received by E-mail. A new job is created within SeedStor and associated with the customer, and their details, if new, are added to the database. The requested material is then documented and our ability to supply these accessions is verified against current stock levels. Once the requested material is finalized, and this can occasionally involve dialog with the customer, it is then picked and packaged ready for dispatch. At this point, MTA paperwork is prepared, phytosanitary requirements are checked and Plant Health Inspections are undertaken as required. For collections that require a cost recovery element, this is processed and an invoice raised. Once all paperwork has been processed to completion, the seed is then sent out by courier to the customer. SeedStor then prompts GRU staff to verify receipt after an expected delivery time and then 6 months later to follow-up for feedback from the customer.) Occasionally clients request variations in the MTA that we are sometimes able to approve and thus new client-specific MTAs are created and uploaded into SeedStor. These are associated with a known client, and from that point on SeedStor can present this MTA as the new standard MTA for these clients for future job requests. SeedStor can also audit the MTA records to ensure those that need to be signed prior to release of material have been received and signed, and indicate any job requests that need to be chased for MTA, or other official documentation (Fig. 4C).

*Regeneration*. Each year the GRU regenerates >2,000 lines across all the collections based on stock levels, age of stocks, demand for specific lines and reported issues with germination or knowledge from failed regenerations in previous growing seasons. This information is entered into SeedStor, thus allowing the system to select the best candidate for regeneration in the field and glasshouse each year based on curator-decided criteria and latest stock information. SeedStor is also used to monitor the progress of all crops during the regeneration so we can monitor losses and add additional phenotype information. This allows us to maintain a clear oversight of each year’s regeneration cycle (Fig. 4D).

## Development of SeedStor

SeedStor was developed following a rigorous evaluation and analysis of available Germplasm Curation tools undertaken in 2014 that demonstrated the need for a fully bespoke system to manage the workflows required by the GRU. SeedStor was created with two functions: the first is the public database that is designed to be freely accessible for use by all interested parties without the need for registration or to sign in. The Control Panel provision of the management tools were bespoke to the GRU and thus located on a different server and required a local authorized user account to function. We have, however, also worked with a small number of other institutes who have received, under license, a standalone copy of the full SeedStor package for their evaluation and use on their own germplasm resources.

### IT infrastructure

SeedStor is hosted on (virtual) servers maintained by the NBIP Computing Department and were built on a typical client-server system using the CiS standard image with Linux (CentOS release 6.9, 64-bit) as the operating system, Apache HTTP server (version 2.2.15) as the Web server, MySQL (version 5.1.73) as the relational database management system, PHP (version 5.3.3) for the server-side processing, and JavaScript for the client-side processing. To implement rich UI applications, JavaScript libraries: jQuery (http://jquery.com) and D3 (http://d3js.org) were used in addition to Bootstrap (http://getbootstrap.com/) and PHPMailer (https://github.com/PHPMailer/PHPMailer) to provide additional functionality.

Presently the SeedStor system resides on two servers to host the Germplasm management system and database: one internal system with the full SeedStor system and a second external server hosting an abridged database containing the publicly accessible information. Each server is configured with 2^Gbyte RAM and 2 vCPUs. Data storage requirements are currently 30^Gbyte, and growth is anticipated at 5^Gbyte per annum, with the majority used by imported photographs of accessions regenerated that year.

### Initial data migration

The collections held by the GRU were built up over many decades, and thus the information connected to these collections was maintained in a wide range of formats from simple paper documents and index cards through to Microsoft Excel spreadsheets and Access databases. The length of time between the earliest samples of a collection and the most recent additions also means that the syntax used to describe material has changed over time and thus a major undertaking is the curation of these data into consistent syntax that can be used within SeedStor. Over time, the GRU has transcribed data from paper to electronic documents; and then from spreadsheets to multi-user databases. This most recent transition to a single MySQL database has required a large investment of time in curating data, cross-checking and identifying discrepancies, and in part explains the time required to bring all of the metadata for material currently held by the GRU into SeedStor to make them publicly available. At this time, we would estimate that the process is about 60% complete.

### Access to bulk data downloads

The results from many of our different search methodologies can also be downloaded as a CSV file for further interrogation prior to requests being made for seed stocks. This includes entire collections through to the results from phenotype searches. Furthermore, the management function allows greater functionality in data export, including the ability to extract data across multiple tables within the database that may not be included within the public server.

### Training, outreach and feedback

SeedStor provides a simple service, the accessibility of seed stocks, using an extensive range of different methods for selecting germplasm material to request. Within the online documentation, we have provided some training resources and have run training sessions during a number of events hosted by the JIC, for example the Wisp Course on Wheat Genetics, 2015. The website provides direct E-mail contact with both GRU curators and the web mastering team (now carried out by the CiS group) for feedback. We have recruited a small group of users from across the primary user groups to test out and provide objective feedback on the system. This group also provides suggestions for new functions and comments on additional functionality or refinements suggested by others that could improve the system. This has resulted in invaluable feedback on several aspects relating to screen layout and formatting which we have incorporated.

### Images

SeedStor contains a large collection of photographs of accessions growing, both in the field and in glasshouses, which can be used visually to identify specific features and phenotypic traits of these accessions. At this time, there are >5,000 images in the system, and this is increasing with every year of regeneration when GRU staff take photographs of all accessions grown that year. This process is automated; once GRU staff rename image files to match the SeedStor StoreCode, they are then bulk uploaded to the server, and SeedStor will correctly associate and move the images to the correct accessions.

## Discussion

The SeedStor database system has greatly improved public access to the accessions and associated electronic data available from the GRU, and the internal management functions have provided a robust workflow to enabling the provision of these materials to research and the general public around the world. Since the introduction of SeedStor, and other initiatives as part of the BBSRC National Capability Grant (NCG), the GRU is processing approximately twice the number of requests for germplasm material each year, compared with before the NCG status, and has a much stronger outreach presence.

We continue to add more material to the SeedStor database including all new collections as they are received by the GRU, as well as historic collections that we hold once fully curated by GRU staff.

The GRU are willing to discuss with other Seed Banks and institutions with a germplasm curation requirement around the world about SeedStor and can provide standalone demonstrations of the system if requested.

## Funding

This research was funded through the Biotechnology and Biological Sciences Research Council (BBSRC) National Capability Grant [BB/IO15280/1] and by the NBI Computing infrastructure for Science (CiS) group.

## Disclosures

The authors have no conflicts of interest to declare.

## Abbreviations

BBSRC: Biotechnology and Biological Sciences Research Council
CiS: Computing infrastructure for Science
GRU: Germplasm Resources Unit
ITPGRFA: International Treaty for Plant Genetic Resources for Food and Agriculture
JIC: John Innes Centre
MLS: Multilateral System
MTA: Material Transfer Agreement
NBIP: Norwich Biosciences Institute Partnership
NGC: National Capability Grant
SSD: single seed descent
WPGSC: Wheat Precise Genetic Stocks Collection
